# Evaluation of Efficacy and Safety for Kanglaite Injection in the Control of the Malignant Pleural Effusions via Thoracic Perfusion: A Systematic Review and Meta-Analysis of Randomized Controlled Trials

**DOI:** 10.3389/fphar.2021.694129

**Published:** 2021-11-03

**Authors:** Guanghui Zhu, Xinmiao Wang, Jie Li, Ying Zhang, Ruike Gao, Xiaoxiao Zhang, Bowen Xu, Jiaqi Hu, Minghao Dai, Jiayang Chen

**Affiliations:** ^1^ Guanganmen Hospital, China Academy of Chinese Medical Sciences, Beijing, China; ^2^ Graduate school, Beijing University of Chinese Medicine, Beijing, China; ^3^ School of Pharmacy, Peking University Health Science Center, Beijing, China; ^4^ Traditional Chinese Medicine Department, Cancer Hospital Chinese Academy of Medical Sciences, Beijing, China

**Keywords:** kanglaite injection, malignant pleural effusion, pleural perfusion, chemotherapeutic drugs, meta-analysis

## Abstract

**Background:** Kanglaite injection (KLTI) is a traditional Chinese medicine (TCM) preparation with anti-tumor activity, which has been used to treat malignant tumors in China. The purpose of this study was to evaluate the efficacy and safety of intrapleural infusion with KLTI in the treatment of malignant pleural effusion (MPE).

**Methods:** Randomized controlled trials (RCTs) on the efficacy and safety of intrathoracic infusion with KLTI in the treatment of MPE were searched from the PubMed, EMBASE, the Cochrane Library, CNKI, VIP, Wanfang and CBM databases. The primary outcome was objective remission rate (ORR). Secondary outcomes included quality of life (QOL) and incidence of adverse events (AEs). The Stata15.1 software and RevMan5.3 software were used to calculate risk ratios (RR) at 95% confidence intervals (CI) and conduct the meta-analysis.

**Results:** This meta-analysis included 20 RCTs, involving 1,291 patients. The ORR of intrapleural infusion with KLTI + chemotherapy drugs in the treatment of MPE was higher than that of chemotherapy alone (RR) 1.23; 95%CI; 1.11–1.36, *I* ^2^ = 0%, *z* = 3.876, *p* = 0.000]. When KLTI is combined with cisplatin or KLTI 200 ml is used in every time, it is more advantageous to improve ORR. Moreover, compared with intrapleural infusion of chemotherapy drugs alone, KLTI combined with chemotherapy drugs significantly improved the QOL of patients with MPE (RR 1.28; 95%CI; 1.70–1.53, *I* ^2^ = 0%, *z* = 2.70, *p* = 0.007). In addition, the participation of KLTI reduced the gastrointestinal reaction (RR 0.79; 95% CI; 0.66–0.96; *I* ^2^ = 0%, *z* = 2.37, *p* = 0.018) and renal damage (RR 0.468; 95% CI; 0.23–0.945, *I* ^2^ = 0%, *z* = 2.11, *p* = 0.035) caused by chemotherapy drugs, but did not increase other adverse reactions (*p* > 0.05).

**Conclusion:** The efficacy and safety of traditional chemotherapy drugs plus KLTI was superior to traditional chemotherapy drugs alone via intrapleural injection in controlling MPE, which suggested that KLTI can be used to treat MPE. However, a more rigorous RCT should be designed and completed before it is widely recommended.

## Introduction

Malignant pleural effusion (MPE), a common complication of advanced malignant tumor, is made by malignant tumor involving pleura or pleural primary tumor. (Heffner, 2008). The aggravation of MPE will lead to obvious dyspnea, which will affect the quality of life (QOL) of patients, and even shorten the survival time. With the prolongation of survival time of patients with malignant tumor, the incidence of MPE gradually increased (Zarogoulidis et al., 2013). Although anti-tumor therapy continues to develop, the preventive and therapeutic effect of MPE is not satisfactory. Some studies have shown that MPE is closely related to shorter survival time. Lung cancer patients with MPE have a survival time of only 5.5 months (Porcel et al., 2015), and patients with other types of cancer with MPE have an overall survival time of 3–12 months (Roberts et al., 2010).

In China, traditional Chinese medicine (TCM) has been used for thousands of years. Supported by modern evidence-based evaluation methods and basic research evidence, it shows that TCM has obvious advantages in anti-tumor and reducing side effects, which is widely used as an alternative or combined treatment of cancer (Wang et al., 2021; Zhang et al., 2021). Kanglaite injection (KLTI), extracted from Coix seed, is one of the most popular anti-cancer TCM injections in China (NCI Dictionary, 2019). In 1995, KLTI was approved by China’s State Food and Drug Administration (drug approval number: Z10970091) for cancer treatment and widely used in anti-tumor therapy (China Food and Drug Administration, 2019; Liu et al., 2019; Li et al., 2020). Since 2002, a series of KLTI clinical studies (NCT00733850, NCT00031031) for cancer patients have been approved and carried out in the United States (Clinicaltrials.gov., 2019). KLTI has been approved by the Ministry of Health of the Russian Federal government as a prescription drug for the treatment of cancer in Russia. Clinical trials have showed that KLTI combined with chemotherapy can better delay the progression of tumor patients, improve disease control rate, prolong survival time and reduce toxicity (Schwartzberg et al., 2017; Lian et al., 2006). Basic studies have also shown that KLTI has a variety of anticancer-related activities, such as promoting apoptosis, destroying the mitotic process, anti-multidrug resistance of tumor cells and improving cellular immunity (Yang et al., 2018; Chen et al., 2021).

Intrapleural perfusion is still one of the main methods for the treatment of MPE, which can increase local drug concentration, cause chemical inflammatory reaction between visceral pleura and mural pleura, stimulate pleural cells to produce mesothelial fibrosis, promote pleural adhesion, reduce pleural permeability and reduce pleural effusion exudation, so as to achieve the purpose of controlling pleural effusion (Scherpereel et al., 2010). However, the adverse reactions caused by intrapleural perfusion of chemotherapeutic drugs are difficult for patients to tolerate, affecting their QOL, so not all MPE patients can benefit from it (Biaoxue et al., 2016). At present, some studies have found that KLTI combined with chemotherapy drugs for intrapleural perfusion has better efficacy than chemotherapy drugs alone, and the incidence of adverse reactions is low. However, the sample sizes of those most clinical studies were small and their quality were low. There was no systematic evaluation method for the efficacy of this therapy to do further evidence-based medicine support. In this study, meta-analysis was used to integrate the clinical research literature of intrapleural perfusion with chemotherapy drugs plus KLTI in the treatment of MPE, in order to provide evidence-based medical evidence for enriching the clinical application of MPE. No similar research topics were found in the PROSPERO website based on the search strategy “(Malignant Pleural Effusion) AND (Kanglaite).”

## Methods

This study was conducted in accordance with the Cochrane Handbook on Systematic Review of Interventions and presented in accordance with the Preferred Reporting Items for Systematic Review and Meta-Analysis (PRISMA) Guidelines (Liberati et al., 2009) (Supplementary Table S1).

### Identification of Literature

We searched and identified the relevant RCTs from the PubMed, EMBASE, the Cochrane Library, CNKI, VIP, Wanfang, and CBM databases (from the establishment of the database to January 2021). The keywords used in the search were as follows: (((“Pleural Effusion, Malignant” (Mesh)) OR (Malignant Pleural Effusion*(Title/Abstract])) OR (MPE (Title/Abstract))) AND ((Kanglaite (Title/Abstract)) OR (Coix Seed Oil (Title/Abstract))) (Supplementary Table S2). In addition, if we found that the references included in the study were closely related to intrapleural perfusion with KLTI, we should further search and identify them. The retrieved research was considered as a potential source and is reviewed manually. Only Chinese and English documents were included.

### Data Variables of Studies

The data we extracted from the original literature are as follows: 1) the year of publication, first author, country of publication; 2) the design type, random mode and implementation method of the study; 3) the number of cases studied and their age, sex and pathological type of tumor and 4) drugs, dosage, course of treatment and treatment time. According to Response Evaluation Criteria in Solid Tumors (RECIST), the outcome data in this meta-analysis included objective remission rate (ORR), Karnofsky score (KPS) and incidence of adverse reactions. The changes of tumor condition include complete response (CR), partial response (PR), stable disease (SD) and progressive disease (PD). ORR was defined as CR + PR.

### Inclusion Criteria of the Study

1) The study design is limited to RCTs comparing traditional chemotherapy drugs plus KLTI (Treatment group) with chemotherapy alone (Control group) in the treatment of MPE; 2) the subjects of MPE must be diagnosed pathologically and/or cytologically and 3) the outcomes must be determined according to RECIST, and the QOL changes must be assessed by KPS.

### Exclusion Criteria of the Study

1) Animal experiments, reviews and other unrelated studies; 2) patients also receiving treatment drugs outside the regimen; 3) no detailed data on ORR, QOL and adverse reactions, or no indicators for them; 4) lack of comparable control groups and 7) single-arm study.

### Supervision of the Implementation Process

The test design must meet the following rules: 1) RCTs of traditional chemotherapy drugs plus KLTI versus traditional chemotherapy drugs alone via intrapleural injection for controlling MPE; 2) the dosage of KLTI was determined by the suggestions of producers, usually 100 ml or 200 ml; 3) number of times of administration: more than or equal to 2 times and 4) observations on efficacy and safety: The main efficacy index was ORR. Subgroup analysis was conducted according to whether combined with cisplatin and the dose of KLTI, in order to clarify the different efficacy of KLTI in each subgroup. Secondary measures included QOL and incidence of adverse events (AEs). AEs included gastrointestinal reactions, myelosuppression, chest pain, fever and renal damage.

### Assessment for Quality of Randomized Controlled Trials

For each included study, two investigators (XMW and RKG) completed the Jadad scale used specifically for the quality of the evaluation method. A third-party researcher (XXZ) was consulted whenever there was a disagreement between the two investigators. The evaluation criteria provided in the Cochrane manual were used to evaluate the quality of the included studies. It includes the following items: 1) random sequence generation (selection bias); 2) blinding of participants and personnel (performance bias); 3) allocation concealment (selection bias); 4) incomplete outcome data (attrition bias); 5) selective reporting (reporting bias) and 6) other bias. According to the above criteria, the quality of the trial is divided into three levels: low bias risk, unclear bias risk, and high bias risk (Xu et al., 2016).

### Statistical Methods and Analysis

Stata 15.1 software was used for this meta-analysis. The risk ratios (RR) was used to analyze the binary variables. By calculating the Z value of chi-square test, *p* < 0.05 is considered to have significant difference. Chi-square (X^2^) test was used for heterogeneity test, and heterogeneity was quantitatively estimated with I-squared (I^2^). If *I* ^2^ ≤ 50% and *p* ≥ 0.1, the homogeneity is good; if heterogeneity exists, the random-effects model would be used to address it. If the heterogeneity test is obvious (*I* ^2^ > 75%), meta regression and subgroup analysis should be considered to explore the source of heterogeneity. *p* values less than 0.05 were considered statistically significant. We use funnel chart to determine publication bias.

## Results

### Literature Retrieval Process

**FIGURE 1:**
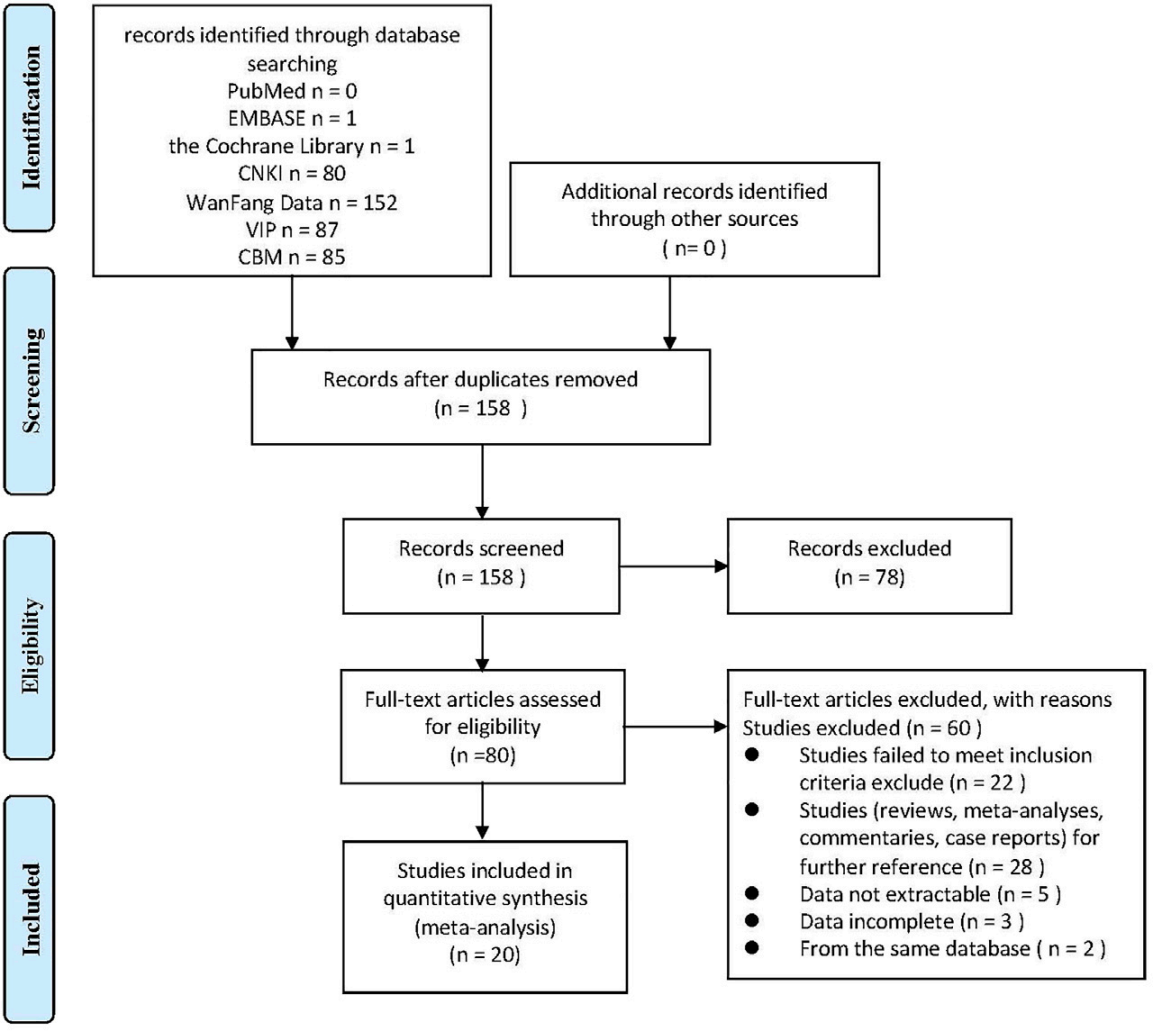
Flowchart of article selection process.

We conducted a systematic search from the database and obtained a total of 406 potential related original studies. 248 repetitive literatures were selected and deleted, the titles and abstracts of the remaining 158 literatures were browsed. 78 of the studies were deleted because the design and implementation of them did not conform to our research theme. After reading the full text of the literature for further screening and evaluation, 60 articles were excluded, including studies that did not meet the inclusion criteria, not belong to clinical studies, the data being incomplete or not be extracted, and from the same database. Finally, a total of 20 original studies were included in the meta-analysis. The flow chart showing the selection process was presented in Figure 1.

### General Characteristics of Included Studies

The 20 original studies (Xie and Zhou, 2000; Zhao et al., 2002; Yang et al., 2005; Jia et al., 2005; Li and Cai, 2005; Jia et al., 2007; Zhang et al., 2009; Chen et al., 2010; Xiao et al., 2011; Xu et al., 2012; Lan et al., 2012; Liu and Zhang, 2012; Li et al., 2012; Wang et al., 2013; Liu et al., 2014; Liu et al., 2016a; Li et al., 2016; Liu et al., 2016b; Wang et al., 2018; Mo et al., 2019) included were all RCTs, all of which were conducted in China, including a total of 1,291 patients. The sample size of the study ranges from 26 (Zhao et al., 2002) to 120 (Liu et al., 2016b). One of the studies did not report the sex and age distribution of the patients (Yang et al., 2005). Patients in other studies were between 21 (Xu et al., 2012) and 87 (Jia et al., 2005) years old, and there were more male (733) than female (520). In all studies, malignant tumors were diagnosed by pathology or pleural effusion cytology. Lung and breast cancer were the most common causes of MPE in the included studies. Table 1 lists the basic information in 20 studies.

**TABLE 1:** Data analysis of included studies.

Author	Year	Region	N (T/C)	Histology of cancer	End point^b^
Xie	2000	China	43 (22/21)	Lung cancer (squamous cell carcinoma, adenocarcinoma, small cell carcinoma), breast cancer, malignant lymphoma	①③⑦
Zhao	2002	China	26 (12/14)	Lung cancer (squamous cell cancer, adenocarcinoma), esophageal cancer, breast cancer	①
Yang	2005	China	50 (27/23)	Lung cancer, breast cancer, prostate cancer, pleural mesothelioma, gastric cancer	①
Jia	2005	China	48 (25/23)	Lung cancer, breast cancer, pleural mesothelioma, stomach cancer, esophageal cancer, kidney cancer	①③④⑦
Li	2005	China	56 (27/29)	Lung cancer, malignant lymphoma, breast cancer	①③
Jia	2007	China	60 (30/30)	Lung cancer, breast cancer, malignant lymphoma	①③④⑥⑦
Zhang	2009	China	34 (20/14)	Lung cancer, breast cancer, malignant lymphoma, ovarian cancer, pleural mesothelioma, bladder cancer, melanoma	①②③⑤⑦
Chen	2010	China	48 (25/23)	Non-small cell lung cancer	①③④⑤⑦
Xiao	2011	China	68 (38/30)	Non-small cell lung cancer	①
Xu	2012	China	48 (24/24)	Lung cancer (adenocarcinoma, squamous cell carcinoma, small cell carcinoma)	①③④⑤
Lan	2012	China	42 (21/21)	Lung cancer, breast cancer, malignant lymphoma	①
Liu	2012	China	64 (34/30)	Lung squamous cell carcinoma, metastatic carcinoma, malignant pleural mesothelioma	①
Li	2012	China	60 (30/30)	Lung cancer (squamous cell carcinoma, adenocarcinoma, adenocarcinoma)	①②
Wang	2013	China	63 (32/31)	Lung cancer, esophageal cancer, breast cancer, malignant lymphoma	①②④
Liu	2014	China	100 (50/50)	Lung cancer, breast cancer, stomach cancer	①②③④
Liu	2016a	China	100 (50/50)	Non-small cell lung cancer	②
Li	2016	China	75 (40/35)	Lung cancer	①②③④⑤⑥⑦
Liu	2016b	China	120 (60/60)	Lung cancer, breast cancer, malignant lymphoma, others	①③④⑤⑥⑦
Wang	2018	China	80 (40/40)	Lung cancer	①②③④⑤⑥⑦
Mo	2019	China	108 (54/54)	Lung cancer (squamous cell carcinoma, adenocarcinoma)	①③④⑤⑦

N number of patients, T Treatment group, C Control group.

a The age of every study is filled in according to the data format reported in the article.

b End point: ①Objective response rate, ②Karnofsky score, ③Gastrointestinal reactions, ④Myelosuppression, ⑤Chest pain, ⑥Renal damage, ⑦Fever.

There were 14 studies (Xie and Zhou, 2000; Jia et al., 2005; Li and Cai, 2005; Yang et al., 2005; Jia et al., 2007; Zhang et al., 2009; Chen et al., 2010; Lan et al., 2012; Li et al., 2012; Liu and Zhang, 2012; Xu et al., 2012; Wang et al., 2013; Li et al., 2016; Mo et al., 2019) of intrapleural infusion of platinum chemotherapeutic drugs and KLTI in the treatment of MPE, of which 10 studies (Xie and Zhou, 2000; Yang et al., 2005; Jia et al., 2005; Li and Cai, 2005; Jia et al., 2007; Zhang et al., 2009; Chen et al., 2010; Lan et al., 2012; Liu and Zhang, 2012; Li et al., 2012; Li et al., 2016) used cisplatin, two studies (Xu et al., 2012; Wang et al., 2013) used carboplatin, and two studies used lobaplatin (Mo et al., 2019) and oxaliplatin (Li et al., 2016) respectively. In addition, four studies used cisplatin + doxorubicin (Zhao et al., 2002), hydroxycamptothecin (Xiao et al., 2011), docetaxel (Liu et al., 2014) or recombinant human interleukin-2 (Mo et al., 2019), and arsenite was used in two studies (Liu et al., 2016a; Liu et al., 2016b). The dosage of KLTI was 100 ml or 200 ml each time, and at least once. Pleural perfusion was performed after pleural effusion drainage. There was no significant difference in baseline data between the two groups (*p* > 0.05), indicating that they were comparable. Table 2 lists the treatments in 20 studies.

**TABLE 2:** Assessment method of administration of included studies.

Author	Year	N (T/C)	Interventions (Groups)		Termination of treatment
			T	C	
Xie	2000	43 (22/21)	KLTI 100 ml 1/week	Cisplatin 50 mg/m^2^ 1/week	≥2 weeks, or MPE disappeared
			Cisplatin 50 mg/m^2^ 1/week		
Zhao	2002	26 (12/14)	KLTI 200 ml 1/week	Cisplatin 50 mg 1/week	≥3 weeks, or MPE disappeared
			Cisplatin 50 mg 1/week	Adriamycin 20 mg 1/week	
			Adriamycin 20 mg 1/week		
Yang	2005	50 (27/23)	KLTI 200 ml 1/week	Cisplatin 60 mg 1/week	≥1 week, or MPE disappeared
			Cisplatin 60 mg 1/week		
Jia	2005	48 (25/23)	KLTI 100 ml 1/week	Cisplatin 50 mg/m^2^ 1/week	2 weeks, or MPE disappeared
			Cisplatin 50 mg/m^2^ 1/week		
Li	2005	56 (27/29)	KLTI 200 ml 1/2∼3days	Cisplatin 60 mg 1/2–3 days	8 days (mean), or MPE disappeared
			Cisplatin 60 mg 1/2–3 days		
Jia	2007	60 (30/30)	KLTI 100 ml 1/week	Cisplatin 40 mg 1/week	≥4 weeks, or MPE disappeared
			Cisplatin 40 mg 1/week		
Zhang	2009	34 (20/14)	KLTI 100 ml	Cisplatin 40 mg	≥4 weeks, or MPE disappeared
			Cisplatin 40 mg		
Chen	2010	48 (25/23)	KLTI 200 ml 1/week	Cisplatin 60 mg 1/week	≥1 week, or MPE disappeared
			Cisplatin 60 mg 1/week		
Xiao	2011	68 (38/30)	KLTI 100 ml 1/week	Hydroxy camptothecin 4 g 1/week	≥1 week, or MPE disappeared
			Hydroxy camptothecin 4 g 1/week		
Xu	2012	48 (24/24)	KLTI 100 ml 1/week	Carboplatin 400 mg 1/week	2 weeks, or MPE disappeared
			Carboplatin 400 mg 1/week		
Lan	2012	42 (21/21)	KLTI 200 ml 2/week	Cisplatin 60 mg 2/week	≥1 week, or MPE disappeared
			Cisplatin 60 mg 2/week		
Liu	2012	64 (34/30)	KLTI 200 ml 1/week	Cisplatin 50 mg/m^2^ 1/week	2 weeks, or MPE disappeared
			Cisplatin 50 mg/m^2^ 1/week		
Li	2012	60 (30/30)	KLTI 100 ml 1/week	Cisplatin 50 mg 1/week	2 weeks, or MPE disappeared
			Cisplatin 50 mg 1/week		
Wang	2013	63 (32/31)	KLTI 100 ml 1/2 weeks	Carboplatin 200–400 mg 1/2 weeks	2 weeks, or MPE disappeared
			Carboplatin 200–400 mg 1/2weeks		
Liu	2014	100 (50/50)	KLTI 100 ml 1/3 days	Docetaxel 40 mg 1/3 days	≥4 weeks, or MPE disappeared
			Docetaxel 40 mg 1/3days		
Liu	2016a	100 (50/50)	KLTI 200 ml 1/4 days	Arsenious acidlnjection 10 mg 1/4 days	≥8 days, or MPE disappeared
			Arsenious acidlnjection 10 mg 1/4days		
Li	2016	75 (40/35)	KLTI 100 ml 1/week	Oxaliplatin 100 mg 1/week	6 weeks, or MPE disappeared
			Oxaliplatin 100 mg 1/week		
Liu	2016b	120 (60/60)	KLTI 200 ml 1/4 days	Arsenious acidlnjection 10 mg 1/4 days	≥4 weeks, or MPE disappeared
			Arsenious acidlnjection 10 mg 1/4days		
Wang	2018	80 (40/40)	KLTI 200 ml 1/week	Recombinant human interleukin-2 1 million U 1/week	3 weeks, or MPE disappeared
			Recombinant human interleukin-2 1 million U 1/week		
Mo	2019	108 (54/54)	KLTI 100 ml 1/3 weeks	Lobaplatin 30 mg/m^2^ 1/3 weeks	6 weeks, or MPE disappeared
			Lobaplatin 30 mg/m^2^ 1/3 weeks		

N number of patients, T Treatment group, C Control group, KLTI Kanglaite injection, MPE malignant pleural effusion.

### The Assessment of Heterogeneity

**FIGURE 2:**
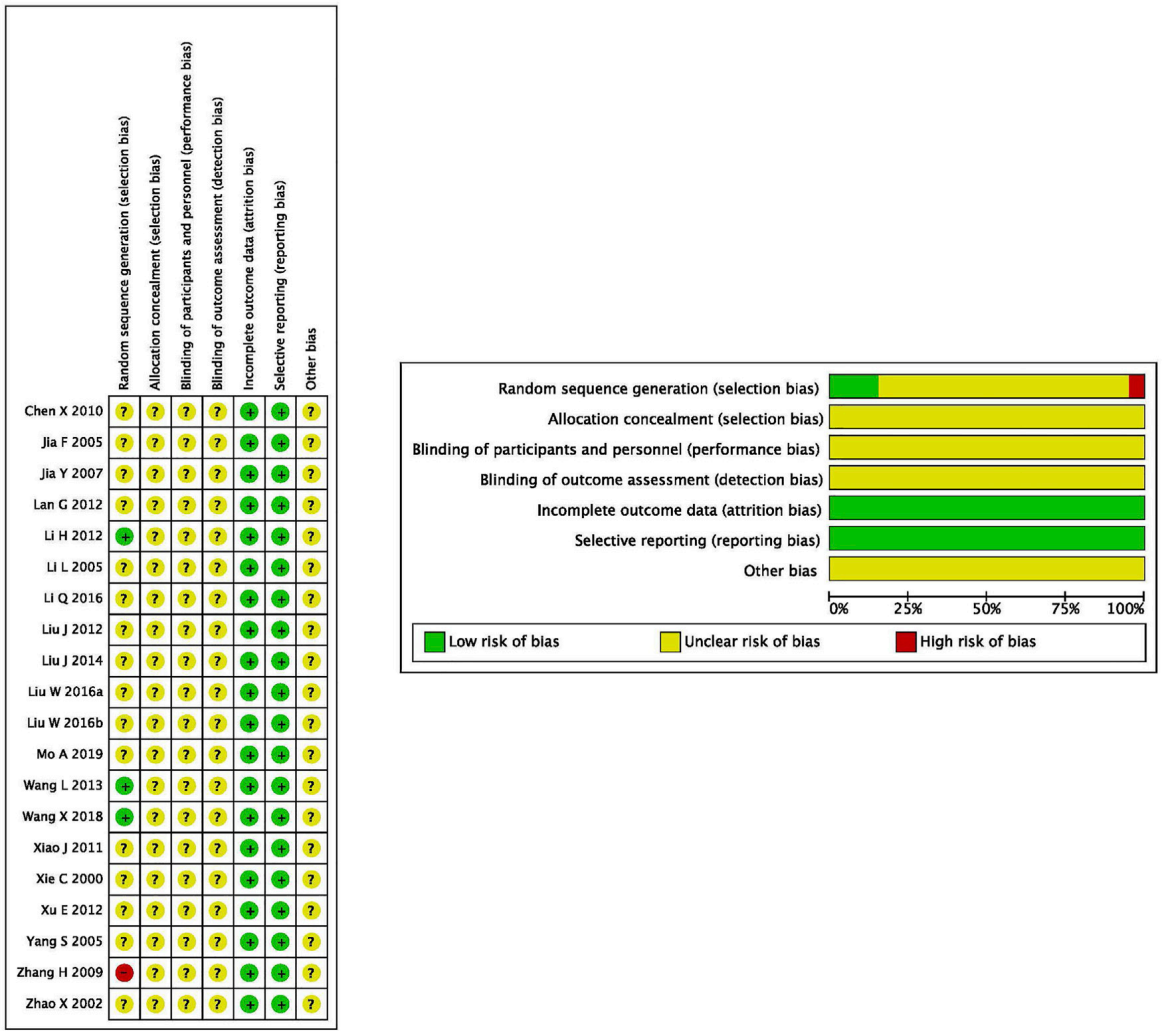
Quality evaluation of included studies: according to the criteria made by the Cochrane Handbook (Version 5.0.1), no heterogeneity existed in eligible RCTs; Overall, these studies had moderate to higher quality.

16 (Xie and Zhou, 2000; Zhao et al., 2002; Yang et al., 2005; Jia et al., 2005; Li and Cai, 2005; Jia et al., 2007; Chen et al., 2010; Xiao et al., 2011; Xu et al., 2012; Liu and Zhang, 2012; Li et al., 2012; Liu et al., 2014; Liu et al., 2016a; Li et al., 2016; Liu et al., 2016b; Mo et al., 2019) of the 20 studies only mentioned the word “random”, so their risk was rated as uncertain. Three studies (Lan et al., 2012; Wang et al., 2013; Wang et al., 2018) were assessed as low risk by using random number table method. One study (Zhang et al., 2009) was randomly assigned according to the time of the patient’s visit, and was assigned alternately, so it was rated as a high risk. No allocation concealment and blind methods were reported in all studies, so the risk was assessed as uncertain. No shedding cases were reported in all studies, and the observation indicators mentioned in the study program were reported in the results. The data of the results are complete and are not selectively reported, so the risk is classified as low. Due to the insufficient information in the literature, other bias risks are classified as uncertainty. (see Figure 2).

## Primary Outcome

### Comparison of Objective Remission Rate Between Traditional Chemotherapy Drugs Plus Kanglaite Injection Versus Traditional Chemotherapy Drugs Alone via Intrapleural Injection for Controlling Malignant Pleural Effusion

**FIGURE 3:**
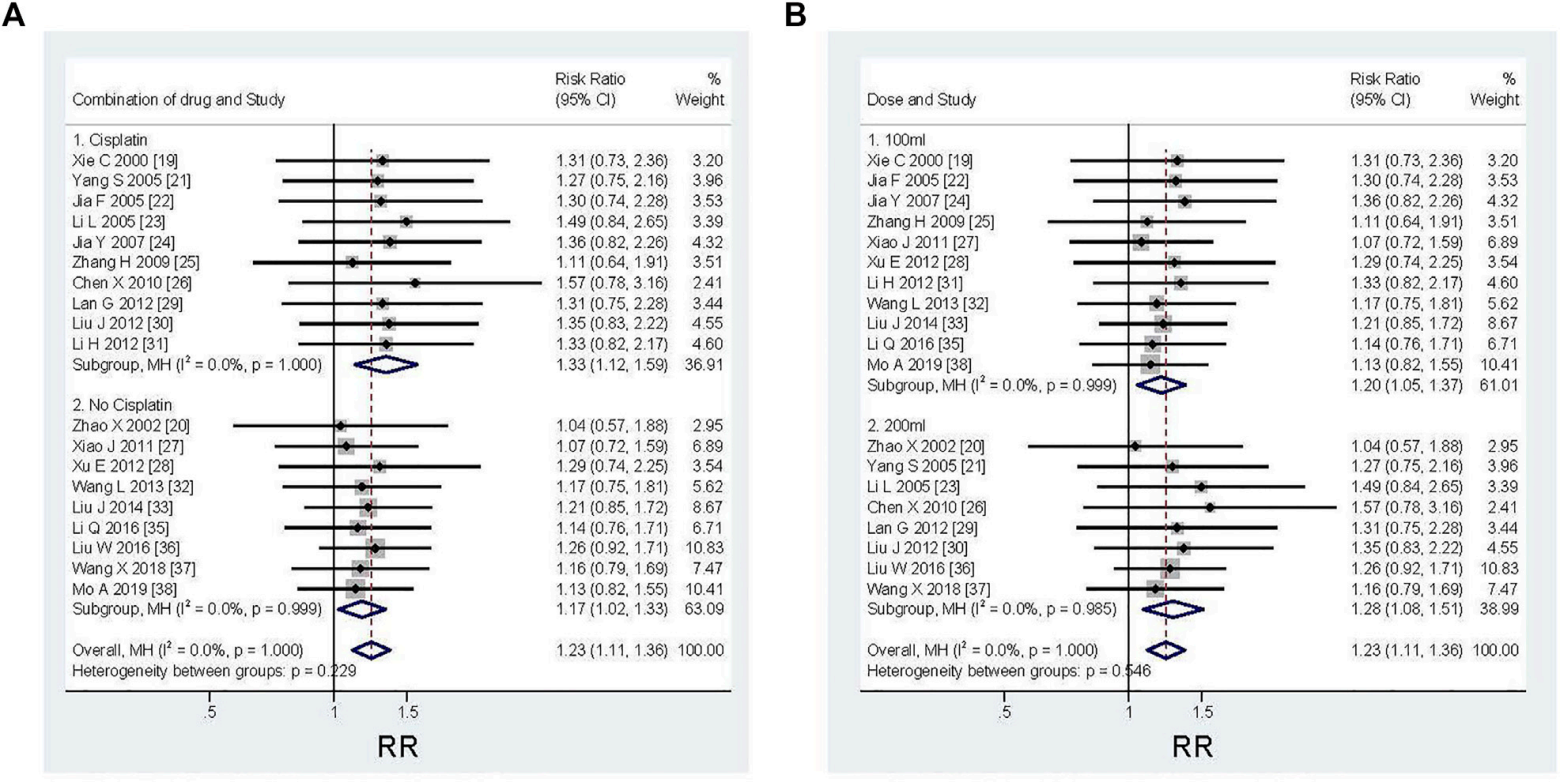
Efficacy comparison of KLTI combined with another agent versus another agent alone by thoracic perfusion for controlling MPE. Subgroup analysis of different types of chemotherapeutic drugs: when combined with cisplatin, the improvement of ORR is more advantageous; Subgroup analysis of different KLTI dosage: the ORR was more advantageous when using KLTI 200 ml each time. RR, Risk ratio; ORR, Objective response rate; KLTI, Kanglaite injection.

Of the 20 studies included, 19 RCTs (Xie and Zhou, 2000; Zhao et al., 2002; Yang et al., 2005; Jia et al., 2005; Li and Cai, 2005; Jia et al., 2007; Zhang et al., 2009; Chen et al., 2010; Xiao et al., 2011; Xu et al., 2012; Lan et al., 2012; Liu and Zhang, 2012; Li et al., 2012; Wang et al., 2013; Liu et al., 2014; Li et al., 2016; Liu et al., 2016b; Wang et al., 2018; Mo et al., 2019) reported ORR data that controlled MPE. The results showed that X^2^ was 3.00 (Degrees of freedom = 18; *p* = 1.000) and that the value of I^2^ (variation in RR attributable to heterogeneity) showed as 0.0%. The results showed that the RR was 1.23 (95%CI; 1.11–1.36, I^2^ = 0%, z = 3.876, *p* = 0.000), which showed that the ORR of traditional chemotherapeutic drugs plus KLTI was a little better than that treated with traditional chemotherapeutic drugs alone (see Figures 3A,B).

In order to explore the efficacy of KLTI combined with different types of chemotherapy drugs in the treatment of MPE, the subgroup analysis showed that ORR could be improved when KLTI combined with cisplatin or non-cisplatin. However, when combined with cisplatin, the improvement is more advantageous (cisplatin versus non-cisplatin: RR 1.33; 95%CI; 1.12–1.59, I^2^ = 0%, z = 3.26, *p* = 0.001 versus RR 1.17; 95%CI; 1.02–1.33, I^2^ = 0%, z = 2.33, *p* = 0.020) (see Figure 3A). According to the subgroup analysis of the dosage of KLTI, it was found that KLTI could both improve ORR, when using 100 ml or 200 ml. But it was more advantageous when using KLTI 200 ml each time (100 versus 200 ml: RR 1.20; 95%CI; 1.05–1.37, I^2^ = 0%, z = 2.64, *p* = 0.008 versus RR 1.28; 95%CI; 1.08–1.51, I^2^ = 0%, z = 2.89, *p* = 0.004) (see Figure 3B).

## Secondary Outcome

### Comparison of Quality of Life Between Traditional Chemotherapy Drugs Plus Kanglaite Injection Versus Traditional Chemotherapy Drugs Alone via Intrapleural Injection for Controlling Malignant Pleural Effusion

**FIGURE 4:**
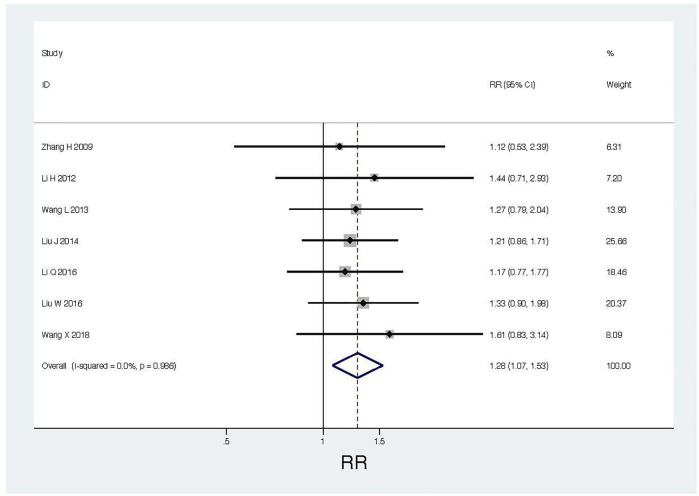
Thoracic perfusion of KLTI combined with other agents improved the QOL of patients with MPE compared with other agents alone. RR, Risk ratio; QOL, Quality of life; KLTI, Kanglaite injection.

QOL improvement was evaluated by the patient’s KPS score. As shown in Table 1, a total of 7 RCTs (Zhang et al., 2009; Li et al., 2012; Wang et al., 2013; Liu et al., 2014; Liu et al., 2016a; Li et al., 2016; Wang et al., 2018) provided KPS data, comparing the QOL between conventional chemotherapy plus KLTI and conventional chemotherapy alone via intrathoracic injection for controlling MPE. After treatment, an increase in KPS score ≥10 was defined as QOL improvement. We found that the improvement rate in the KLTI combined perfusion group (190/262, 72.52%) was higher than that in the simple chemotherapy group (122/250, 48.80%). The heterogeneity analysis showed that X^2^ was 0.99 (Degrees of freedom = 6, *p* = 0.986), I^2^ = 0.0%. The results of meta-analysis showed that the combined RR was 1.28 (95%CI; 1.70–1.53, I^2^ = 0%, z = 2.70, *p* = 0.007), which indicated that intrapleural infusion of KLTI combined with chemical drugs could significantly improve the QOL of patients with MPE compared with chemical drugs alone (see Figure 4).

### Composition Ratio of Adverse Events on Traditional Chemotherapy Drugs Plus Kanglaite Injection Versus Traditional Chemotherapy Drugs Alone via Intrapleural Injection for Controlling Malignant Pleural Effusion

The AEs reported in 12 items of RCTs (Xie and Zhou, 2000; Jia et al., 2005; Li and Cai, 2005; Jia et al., 2007; Zhang et al., 2009; Chen et al., 2010; Xu et al., 2012; Liu et al., 2014; Li et al., 2016; Liu et al., 2016b; Wang et al., 2018; Mo et al., 2019) were gastrointestinal reactions (such as nausea or/and vomiting) (Combined group versus Single drug group: 132/417, 31.65% versus 179/401, 44.64%). 10 items of RCTs (Jia et al., 2005; Jia et al., 2007; Chen et al., 2010; Xu et al., 2012; Wang et al., 2013; Liu et al., 2014; Li et al., 2016; Liu et al., 2016b; Wang et al., 2018; Mo et al., 2019) reported myelosuppression (such as leukopenia, hemoglobin or/and thrombocytopenia) (68/380, 17.89% versus 78/373, 20.91%). Seven RCTs (Zhang et al., 2009; Chen et al., 2010; Xu et al., 2012; Li et al., 2016; Liu et al., 2016b; Wang et al., 2018; Mo et al., 2019) reported chest pain (68/263, 25.86% versus 72/248, 29.03%). Nine RCTs (Xie and Zhou, 2000; Jia et al., 2005; Jia et al., 2007; Zhang et al., 2009; Chen et al., 2010; Li et al., 2016; Liu et al., 2016b; Wang et al., 2018; Mo et al., 2019) reported fever (42/316, 13.29% vs 35/300, 11.67%). Four items of RCTs (Jia et al., 2007; Liu et al., 2016b; Li et al., 2016; Wang et al., 2018) reported renal damage (10/170, 5.88% vs 24/165, 14.55%) (see Table 3).

**TABLE 3:** Comparison of adverse events between KLTI combined with chemotherapeutic agents versus chemotherapeutic agents alone.

Author	Year	Gastrointestinal reactions (%)		Myelosuppression (%)		Chest pain (%)		Fever (%)		Renal damage (%)	
		T	C	T	C	T	C	T	C	T	C
Xie	2000	9 (40.91)	15 (71.43)					4 (18.18)	1 (4.76)		
Zhao	2002										
Yang	2005										
Jia	2005	9 (36.00)	15 (65.22)	2 (8.00)	3 (13.40)			4 (16.00)	1 (4.35)		
Li	2005	12 (44.44)	14 (48.28)								
Jia	2007	7 (23.33)	22 (73.33)	4 (13.33)	7 (23.33)			7 (23.33)	2 (6.67)	4 (13.33)	12 (40.00)
Zhang	2009	11 (55.00)	12 (85.71)			4 (20.00)	10 (71.43)	3 (15.00)	3 (21.43)		
Chen	2010	3 (12.00)	10 (43.48)	7 (28.00)	8 (34.78)	12 (48.00)	4 (17.39)	6 (24.00)	5 (21.74)		
Xiao	2011										
Xu	2012	17 (70.83)	20 (90.91)	15 (62.50)	18 (81.82)	4 (16.67)	3 (13.64)				
Lan	2012										
Liu	2012										
Li	2012										
Wang	2013			5 (15.63)	6 (19.35)						
Liu	2014	15 (30.00)	14 (28.00)	6 (12.00)	6 (12.00)						
Liu	2016a										
Li	2016	14 (35.00)	16 (45.71)	11 (27.50)	9 (22.50)	12 (30.00)	13 (37.14)	2 (5.00)	0 (0)	1 (2.50)	2 (5.71)
Liu	2016b	5 (8.33)	11 (18.33)	3 (5.00)	4 (6.67)	5 (8.33)	12 (20.00)	9 (15.00)	14 (23.33)	2 (3.33)	6 (10.00)
Wang	2018	7 (17.50)	9 (22.50)	3 (7.50)	4 (10.00)	9 (22.50)	10 (25.00)	4 (10.00)	5 (12.50)	3 (7.50)	4 (10.00)
Mo	2019	23 (42.59)	21 (38.89)	12 (2.22)	13 (24.07)	22 (40.74)	20 (37.04)	3 (5.56)	4 (7.41)		

T Treatment group, C Control group, KLTI Kanglaite injection.

### Comparison of Adverse Events Between Traditional Chemotherapy Drugs Plus Kanglaite Injection Versus Traditional Chemotherapy Drugs Alone via Intrapleural Injection for Controlling Malignant Pleural Effusion

Heterogeneity test found that I^2^ ≤ 50% and *p* ≥ 0.1, indicating that homogeneity is good. By comparing the results of AEs, it was found that the incidence of AEs in the treatment group was significantly lower than that in the control group (RR 0.84; 95%CI; 0.73–0.96, I^2^ = 0%, z = 2.64, *p* = 0.008). Among them, the incidence of gastrointestinal reactions (nausea or/and vomiting) in the treatment group was significantly lower than that in the control group (RR 0.79; 95% CI; 0.66–0.96; I^2^ = 0%, z = 2.37, *p* = 0.018). The incidence of renal damage in the treatment group was significantly lower than that in the control group (RR 0.468; 95% CI; 0.23–0.945, I^2^ = 0%, z = 2.11, *p* = 0.035).

**FIGURE 5:**
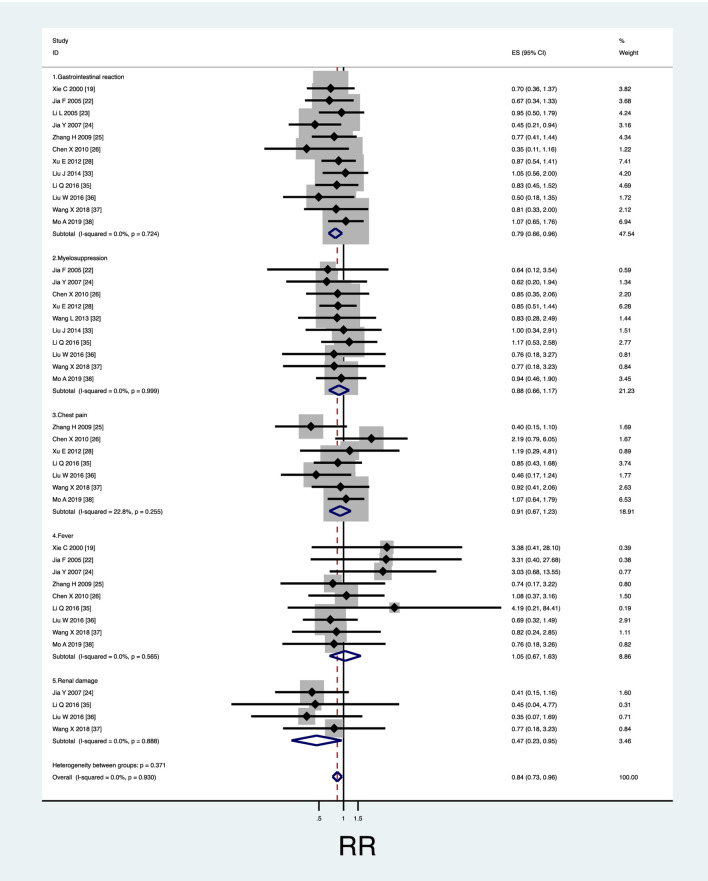
Safety evaluation of KLTI combined with another agent versus another agent alone by thoracic perfusion for controlling MPE. **(A)** The KLTI combination therapy displayed a lower incidence rate of gastrointestinal reactions than the project of other agents alone; **(B)** No difference in incidence rate of myelosuppression was testified between KLTI combined with other agents and other agents alone; **(C)** No difference in incidence rate of chest pain was testified between KLTI combined with other agents and other agents alone; **(D)** No difference in incidence rate of fever was testified between KLTI combined with other agents and other agents alone; **(E)** No difference in incidence rate of renal damage was testified between KLTI combined with other agents and other agents alone. RR, Risk ratio; KLTI, Kanglaite injection.

In addition, there was no significant difference in the incidence of myelosuppression (RR 0.88; 95% CI; 0.66–1.17, I^2^ = 0%, z = 0.88, *p* = 0.377), chest pain (RR 0.91; 95% CI; 0.67–1.23, I^2^ = 22.8%, z = 0.63, *p* = 0.532) and fever (RR 1.047; 95% CI; 0.674–1.628, I^2^ = 0%, z = 0.20, *p* = 0.838) between the two groups (see Figure 5).

### Assessment of Publication Bias

**FIGURE 6:**
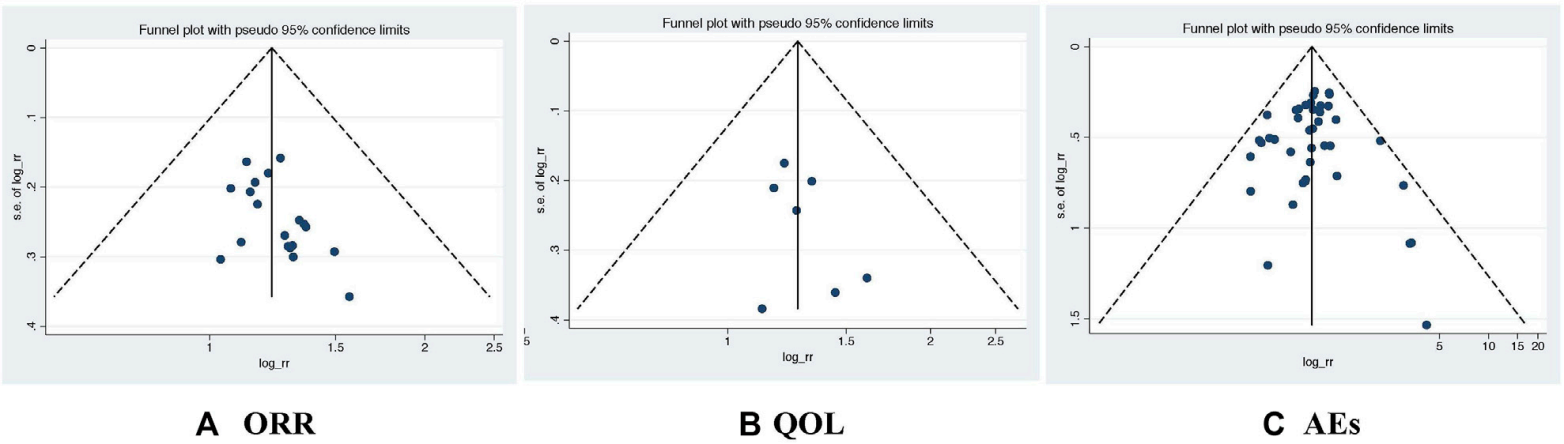
The shape of the funnelplot appeared to be approximately symmetrical. ORR, Objective response rate; QOL, Quality of life; AEs, Adverse events.

We drew a funnel chart and noticed that the included study was symmetrically distributed on both sides of the funnel chart (see Figures 6A–C), indicating that there was no significant publication bias in the meta-analysis.

## Discussion

KLTI is a kind of TCM preparation with bi-directional broad-spectrum anticancer effect, which is the extract of coix seed. Its main chemical component coixenolide has been proved to be an effective anticancer ingredient. Basic research found that KLTI can inhibit tumor cell proliferation and induce its apoptosis. Jiang Y et al. (Jiang et al., 2015) found that for A549 and H1975 NSCLC cell lines, KLTI mainly blocked cells in G2-M phase and combined with pemetrexed in S phase to enhance the cytotoxicity of chemotherapy. In addition, KLTI also plays a role in inhibiting cancer cell migration and intratumoral angiogenesis (Woo et al., 2007; Jiang and Fang, 2019). KLTI can regulate immune function. Pan et al. (Pan et al., 2012) found that KLTI can increase the level of serum IL-2 and stimulate T cell proliferation in Lewis lung cancer-bearing mice. In the study of Kunming lung cancer mice by Duan (Duan, 2018), it was found that compared with cisplatin alone, KLTI combined with cisplatin could significantly increase the spleen index and enhance the immune function of mice by reducing the level of TAM. A large number of clinical studies have shown that KLTI is effective in enhancing anti-tumor efficacy, improving patients’ quality of life and reducing adverse reactions (Huang et al., 2020; Li et al., 2020).

Through systematic search and screening, we finally included 20 items of RCTs for this meta-analysis to compare the efficacy and safety of KLTI combined with chemotherapy and chemotherapy alone in the treatment of MPE in the form of thoracic perfusion. Through statistical verification and combined with the clinical information of those studies, we found that those included RCTs have good homogeneity and comparability. This meta-analysis showed that compared with intrathoracic infusion of traditional chemotherapeutic drugs in the treatment of MPE, KLTI combined with chemotherapeutic drugs could benefit ORR (RR = 1.229, *p* = 0.000). The ORR of patients with MPE treated by intrapleural infusion of KLTI + chemotherapeutic drugs was 1.229 times higher than that of chemotherapeutic drugs alone. The results showed that KLTI played an important role in the thoracic perfusion treatment of MPE, indicating that KLTI could be used as an alternative drug to control MPE in clinical application. Network pharmacological study (Wang, 2019) showed that three main active components were screened from KLTI: triglyceride, coixenin and coixenolide. There are 25 potential anticancer targets of the three components, which can treat 22 kinds of cancer through different pathways. After KEGG analysis, 7 pathways were found to be closely related to the anticancer effect, mainly related to the regulation of cell proliferation, protein kinase B, cyclooxygenase pathway and so on. It can promote the secretion of TNF- α, inhibit the expression of COX-2 and regulate the activity of transcription factor FOX3a. Therefore, KLTI can control the tumor from multi-target and multi-angle.

With the emergence of anti-tumor measures, the survival time of tumor patients is longer than before, so the QOL of patients has been widely concerned by patients, society and doctors. Nowadays, more and more clinical studies use QOL improvement in patients to evaluate the effectiveness of treatment (Anota et al., 2015). According to this meta-analysis, it was found that the improvement of QOL in patients with MPE treated by intrathoracic infusion of KLTI combined with chemotherapeutic drugs was better than that of chemotherapy drugs alone (RR = 1.281, 0.007). The quality of life of patients with intrathoracic infusion of KLTI was 0.281 times higher than that of patients with intrapleural infusion of chemotherapeutic drugs. Previous studies have shown that coix seed can affect energy synthesis by regulating glucose and lipid metabolism, which may be the mechanism of improving the QOL of patients. Kim et al. (Kim et al., 2007) fed the obese rats with the water extract of Coix seed. It was found that the leptin level, fat content and body weight in the experimental group were lower than those in the control group. The expression of neuropeptide Y and leptin receptor was also decreased. The aqueous extract of Coix seed can control the energy balance by regulating the expression of neuropeptide Y and leptin receptor in hypothalamus. Chen et al. (Chen et al., 2019) isolated a water-soluble polysaccharide PAS from coix seed. After using it to treat type 2 diabetic mice, it was found that the content of insulin in mice increased. Oxidative stress and fat accumulation caused by hyperglycemia were also improved. In addition, the immune enhancement function of KLTI can also affect the QOL. Huang Ting et al. (Huang and Li, 2018) believed that KLTI could promote the activity and proliferation of NK cells, and not affect the immune function of normal mice, which may be related to PI3K/AKT pathway. However, it can enhance the immune function of mice at the cellular level and promote the ability of antibody production in mice (Zhou et al., 2018).

At present, chemotherapy is still one of the main anti-tumor measures. However, the adverse reactions caused by chemotherapeutic drugs, such as digestive tract reactions, myelosuppression and renal damage, affect patients’ tolerance to treatment, reduce patients’ quality of life, and may also lead to insufficient treatment cycle. it has become one of the important issues of clinicians. The results of meta-analysis showed that intrapleural infusion of KLTI could significantly reduce the incidence of chemotherapeutic drug-related adverse reactions (RR = 0.838, *p* = 0.008). In the study included in this meta-analysis, it was found that gastrointestinal reactions and myelosuppression were the most common adverse reactions. The results showed that intrathoracic infusion of KLTI could significantly reduce the incidence of digestive tract reactions (RR = 0.79, *p* = 0.018), and did not increase the incidence of myelosuppression (RR = 0.878, *p* = 0.377). In addition, intrathoracic infusion of KLTI reduced the incidence of renal damage (RR = 0.468, *p* = 0.035), but did not increase the burden of chest pain and fever (RR = 0.908, *p* = 0.532; RR = 1.047, *p* = 0.838). It is suggested that intrathoracic infusion of KLTI is safe in the treatment of MPE and can reduce the occurrence of some adverse reactions. Li et al. (Li et al., 2019) found that compared with other traditional Chinese medicine injections, there was no significant statistical difference in the effective rate of intrathoracic infusion of KLTI + cisplatin in the treatment of MPE. However, according to the probability ranking results, the incidence of nausea and vomiting in KLTI + cisplatin regimen was the lowest.

After the heterogeneity test of the results, it is found that the included studies have excellent homogeneity and comparability in the merger of the results. According to the symmetry of funnel chart, no obvious publication bias was found.

However, this meta-analysis still has some limitations:

①most of the samples included in the study are very small, which reduces the efficiency of the test.
②The blind method was not implemented in the study, and whether to implement distributive concealment was not described. The lax research process may exaggerate the efficacy.
③Because KLTI has been approved by China’s State Food and Drug Administration, most of the patients are from China, which may lead to geographical and ethnic differences.
④This study is designed for simple meta-analysis, not reticular meta-analysis, so there is no difference in efficacy between KLTI and other TCM injections.
⑤All the original literatures included were MPE caused by lung cancer. First, because MPE is most common in patients with lung cancer (Penz et al., 2017). In addition, KLTI was registered in China for use in lung and liver cancer. In the future, high-quality RCTs should be designed to evaluate the efficacy of KLTI in the treatment of MPE caused by other cancers. Nevertheless, our results provide an important recommendation that KLTI is effective and safe and can be used in combination with chemotherapeutic drugs for pleural perfusion in the treatment of MPE.

## Conclusion

Compared with chemotherapy alone, intrathoracic infusion of KLTI + chemotherapy drugs can more effectively improve ORR of patients with MPE, and can also improve the QOL of patients. When combined with cisplatin and each use of KLTI 200ml, the effect is more significant. In addition, the participation of KLTI can also reduce the toxicity caused by chemotherapeutic drugs. However, a more rigorous RCT should be designed and completed before it is widely recommended.

## Data Availability

The original contributions presented in the study are included in the article/Supplementary Material, further inquiries can be directed to the corresponding author.

## Abbreviations

KLTI, Kanglaite injection; TCM, Traditional Chinese medicine; MPE, malignant pleural effusion; RCTs, Randomized controlled trials; ORR, Objective remission rate; QOL, quality of life; WHO, World Health Organization; RECIST, Response Evaluation Criteria in Solid Tumors; KPS, Karnofsky score; CR, complete response; PR, Partial response; SD, stable disease; PD, Progressive disease; AEs, Adverse events; RR, risk ratios; X2, Chi-square; I2, I-squared.
