# Supplementary material for: Evaluation of Efficacy and Safety for Kanglaite Injection in the Control of the Malignant Pleural Effusions via Thoracic Perfusion: A Systematic Review and Meta-Analysis of Randomized Controlled Trials
Source: Front Pharmacol. 2021 Nov 3;12:694129. doi: 10.3389/fphar.2021.694129 (PMC8595202; doi:10.3389/fphar.2021.694129)
Supplement: Supplementary file 2 [file Table2.DOCX]

| #4 | Search: **((("Pleural Effusion, Malignant"[Mesh]) OR (Malignant Pleural Effusion*[Title/Abstract])) OR (MPE[Title/Abstract])) AND ((Kanglaite[Title/Abstract]) OR (Coix Seed Oil[Title/Abstract]))** Sort by: **Publication Date** | [0](https://pubmed.ncbi.nlm.nih.gov/?term=%28%28%28%22Pleural+Effusion%2C+Malignant%22%5BMesh%5D%29+OR+%28Malignant+Pleural+Effusion%2A%5BTitle%2FAbstract%5D%29%29+OR+%28MPE%5BTitle%2FAbstract%5D%29%29+AND+%28%28Kanglaite%5BTitle%2FAbstract%5D%29+OR+%28Coix+Seed+Oil%5BTitle%2FAbstract%5D%29%29&sort=pubdate&size=200) | 22:23:57 |
| --- | --- | --- | --- |
| #3 | Search: **(Kanglaite[Title/Abstract]) OR (Coix Seed Oil[Title/Abstract])** Sort by: **Publication Date** | [75](https://pubmed.ncbi.nlm.nih.gov/?term=%28Kanglaite%5BTitle%2FAbstract%5D%29+OR+%28Coix+Seed+Oil%5BTitle%2FAbstract%5D%29&sort=pubdate&size=200) | 22:22:00 |
| #2 | Search: **(("Pleural Effusion, Malignant"[Mesh]) OR (Malignant Pleural Effusion*[Title/Abstract])) OR (MPE[Title/Abstract])** Sort by: **Publication Date** | [6,563](https://pubmed.ncbi.nlm.nih.gov/?term=%28%28%22Pleural+Effusion%2C+Malignant%22%5BMesh%5D%29+OR+%28Malignant+Pleural+Effusion%2A%5BTitle%2FAbstract%5D%29%29+OR+%28MPE%5BTitle%2FAbstract%5D%29&sort=pubdate&size=200) | 22:19:49 |
| #1 | Search: **"Pleural Effusion, Malignant"[Mesh]** Sort by: **Most Recent** | [3,906](https://pubmed.ncbi.nlm.nih.gov/?sort=date&term=%22Pleural+Effusion%2C+Malignant%22%5BMesh%5D&size=200) | 22:19:12 |
